# Effect of fluid preloading on postoperative nausea and vomiting following laparoscopic cholecystectomy

**DOI:** 10.4103/1658-354X.57872

**Published:** 2009

**Authors:** Ahmed Turkistani, Khalid Abdullah, Essam Manaa, Bilal Delvi, Gamal Khairy, Badiah Abdulghani, Nancy Khalil, Fatma Damas, Abdelazeem El-Dawlatly

**Affiliations:** *Department of Anesthesia, College of Medicine, King Saud University, Kingdom of Saudi Arabia*; 1 *Department of Surgery, College of Medicine, King Saud University, Kingdom of Saudi Arabia*; 2 *Department of Anesthesia, National Guard Hospital, Riyadh, Kingdom of Saudi Arabia*

**Keywords:** *Crystalloid*, *colloids*, *postoperative nausea and vomiting*, *general anesthesia*

## Abstract

**Background:**

Postoperative nausea and vomiting (PONV) is a common complication following general anesthesia. Different regimens have been described for the treatment of PONV with few that mention the prevention of it. Therefore, we conducted this study to compare the effect of preloading with either crystalloids or colloids on the incidence of PONV following laparoscopic cholecystectomy (LC), under general anesthesia.

**Materials and Methods:**

This study was carried out on 80 patients who underwent LC. The patients were divided into four groups (each 20 patients), to receive preloading of intravenous fluid, as follows: Group 1 received, 10 ml/kg of low-MW tetrastarch in saline (Voluven™), group 2 received, 10 ml/kg medium-MW pentastarch in saline (Pentaspan™), group 3, received 10 ml/kg of high-MW heta-starch in saline (Hespan™), and group 4, received 10 ml/kg Lactated Ringer's, and this was considered as the control group. All patients received the standard anesthetic technique. The incidence of PONV was recorded, two and 24 hours following surgery. The need for antiemetics and/or analgesics was recorded postoperatively.

**Results:**

The highest incidence of PONV was in group 3 (75% of the patients) compared to the other three groups. Also the same trend was found with regard to the number of patients who needed antiemetic therapy. It was the highest incidence in group 3 (70%), followed by group 2 (60%), and then group 1(35%), and the least one was in the control group (25%).

**Conclusion:**

Intravascular volume deficits may be a factor in PONV and preloading with crystalloids showed a lower incidence of PONV.

## INTRODUCTION

Postoperative nausea and vomiting (PONV) continues to be a common complication after general anesthesia, which can lead to high levels of patient distress and dissatisfaction.[1] Following elective surgery, PONV is believed to result from gut ischemia, secondary to hypovolemia due to overnight fasting. A number of risk factors have been identified for PONV. These include factors relating to the patient, anesthesia, surgical procedure, and postoperative factors.[2] Current approaches for prevention and treatment of PONV remain limited, and 25% of the patients continue to experience PONV within 24 hours of surgery.[3] Despite this, universal pharmacological PONV prophylaxis does not seem to be cost effective, and may be associated with increased side effects. Although some advocate prophylactic antiemetic therapy for high-risk patients, with rescue antiemetic treatment for episodes of PONV, the optimal approach remains unclear.[4] There remains a need to develop cost-effective, ideally non-pharmacological strategies to decrease the incidence of PONV. Perioperative administration of a sufficient volume of intravenous fluids to correct the fasting hours' deficit may effectively prevent PONV, without the expense or the potential for side effects seen with pharmacological approaches.[5] Replacement of assumed preoperative deficits, in addition to a generous substitution of unsubstantiated, increased, insensible perspiration and third space loss, plays an important role in the current perioperative fluid regimens. Perioperative fluid application has been a topic of debate in the past years.[6] Therefore, the potential efficacy of intravenous fluid therapy in reducing PONV remains to be convincingly demonstrated.[7]

The aim of this study is to evaluate the effect of either crystalloids or colloids in different concentrations, for intravenous fluid preloading on the incidence of PONV, on patients undergoing elective LC under general anesthesia.

## MATERIALS AND METHODS

This study was carried out on 80 patients of both sexes (55 females), ASA I-II, who underwent LC under general anesthesia. This prospective randomized, controlled, clinical trial was conducted after approval from the hospital ethical committee and patient's written informed consent. The age of the patients ranged between 19 and 46 years. All the patients were nonsmokers. Patients taking antiemetic drugs, with BMI >30, history of motion sickness, those who experienced nausea and vomiting on the morning of surgery or any patient with documented disease of renal, cardiac, hepatic, nervous or gastrointestinal system (other than gallstones) were excluded from the study. During the preoperative visit, all the patients were familiarized with a visual analog scale (VAS) of 0-10 for PONV.[8] On this scale, score 0 meant no nausea, while score 10 meant the worst imaginable nausea. Occurrence of vomiting was scored as 10. They were also familiarized with the VAS for pain. Pain was scored at regular intervals postoperatively using the VAS scale with 0 as no pain and 10 as the worst imaginable pain. All patients were kept nil per orally eight hours before surgery and were premedicated with oral lorazepam 1-2 mg about two hours prior to surgery.

In the operation theater, an 18-G i.v. cannula was inserted, and monitoring for heart rate, blood pressure, ECG, end-tidal CO_2_, and SpO_2_ was initiated. The patients were randomly allocated to one of the four groups. Thereafter, intravenous fluids were administered to the patients over a period of 15 minutes prior to the induction of anesthesia, in accordance with the groups, as shown below:

**Table:** 

Group 1 (20):	Received 10 ml/kg of low-MW tetrastarch in saline (Voluven™)
Group 2 (20):	Received 10 ml/kg medium-MW pentastarch in saline (Pentaspan™)
Group 3 (20):	Received 10 ml/kg of high-MW hetastarch in saline (Hespan™)
Group 4 (20):	Received 10 ml/kg Lactated Ringer's (LR) (Control Group)

The observer was not present in the operation theater at the time of preloading or during conduction of the case under general anesthesia. Anesthesia was induced only after infusing the full amount of calculated intravenous fluid. After fluid administration, anesthesia was induced using i.v. fentanyl 2 micrograms/kg, propofol 2 mg/kg, and tracheal intubation was facilitated using atracurium 0.5 mg/kg. Anesthesia was maintained using sevoflurane 1-2% and oxygen 50% in air. An orogastric tube was inserted and left on continuous drainage in all the cases. Intraoperatively, LR was given to all the patients at the rate of 6 ml/kg/h. At the end of surgery, muscle relaxation was antagonized using neostigmine 0.05 mg/kg and atropine 0.01 mg/ kg. The orogastric tube was aspirated and then removed prior to extubation of the trachea. The surgical wounds of all patients were infiltrated with 0.25% bupivacaine for postoperative analgesia. Additionally, an i.m. injection of diclofenac sodium 1.5 mg/kg was given eight hourly. No prophylactic antiemetic was given to any patient. The duration of anesthesia was defined as the time from induction to extubation of the trachea. Duration of surgery was defined as the time from surgical incision to closure of skin. All patients received oxygen supplementation (30-40%), using a facemask, for 4 hours, postoperatively. Intravenous fluids were continued in the form of LR (2 ml/kg/h) for 24 hours postoperatively.

The blinded observer made all the observations in the postoperative period. The VAS scores for PONV were recorded on PACU admission, two hours and 24 hours postoperatively. Incidence of PONV from 0-2 hours postoperatively was labeled as ‘early PONV’ and that after two hours was labeled as ‘late PONV’. Injection ondansetron 4 mg i.v was used as a rescue antiemetic whenever the VAS score became >5 or the patient vomited.

### Statistics

Statistical analysis was made using the SPSS version 10. Analysis of variance, student's *t*-test, and binomial analyses were performed as appropriate. The number needed to treat was the reciprocal of the absolute risk reduction between the two groups. Demographic variables were analyzed by one-way analysis of variance. Intergroup comparison of VAS scores (for PONV and pain) were performed using repeated measures of ANOVA. A sample size of 20 in each group had an 80% power to detect a reduction of incidence of PONV by 34% between the control and treatment groups, with a significance level (alpha) of 0.05 (two-tailed)

## RESULTS

Demographic data of all patients, duration of surgery, and anesthesia are given in Table 1.

**Table 1:** Demographic data of the patients (mean ± SD)

	Group 1 Tetrastarch	Group 2 Pentastarch	Group 3 Hetastarch	Group 4 LR
Age (years)	30.70 ± 6.15	33.85 ± 7.23	32.25 ± 6.98	31.80 ± 8.17
Weight (kg)	71.15 ± 14.8	72.4 ± 13	74.45 ± 1401	73.65 ± 18.5
Sex (f/m)	13/7	14/6	14/6	14/6
Duration of surgery (minutes)	86.40 ± 11.37	87.35 ± 16.37	83.45 ± 17.42	86.50 ± 18.61
Duration of anesthesia (minutes)	102.15 ± 10.35	103.50 ± 13.32	99.40 ± 17.65	101.30 ± 12.45

LR - Lactated Ringer

**Figure 1:**
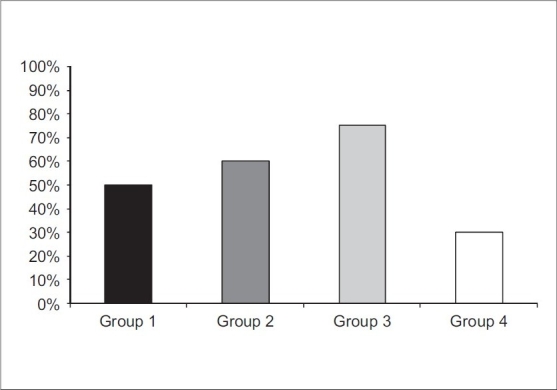
Total percentage of postoperative nausea and vomiting in studied groups

**Figure 2:**
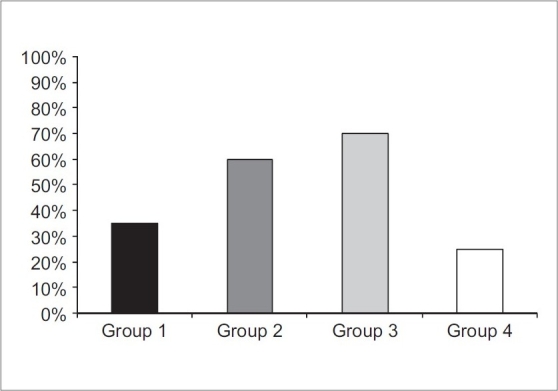
Patients who needed antiemetics

The incidence of PONV was observed as the least percentage in group 4 (30%), which was significant when compared with the other three groups (*P* < 0.05) [Figure 1]. The incidence of PONV at the 24-hour period, postoperatively, was only 5% in group 1 compared to 20, 20, and 15% in groups 2, 3, and 4, respectively [Table 2]. In the same table, the incidence of PONV, two hours postoperatively, was 5% in group 4 compared to 35, 45, and 60% in groups 1, 2 and 3 respectively (*P* < 0.05). The same trend was recorded for patients who needed antiemetic agents for treatment of PONV postoperatively. It was significantly lower (*P* < 0.05) in the control group (25%) compared to the other three groups, wherein it was (35%) in group 1, followed by group 2 (60%), and it was the highest in group 3 (70%) [Table 3 and Figure 2]. There was no significant difference (*P* > 0.05) with regard to patients who needed more doses of analgesics in the four studied groups [Table 4].

**Table 2:** Incidence of postoperative nausea and vomiting in all groups

Group	PACU (%)	2 hours (%)	24 hours (%)	Total percent of PONV (%)
1	25 (5/20)	35 (7/20)	5 (1/20)	50 (10/20)
2	35 (7/20)	45 (13/20)	20 (4/20)	60 (12/20)
3	20 (4/20)	60 (12/20)	20 (4/20)	75 (15/20)
4	15 (3/20)	5 (1/20)	15 (3/20)	30 (6/20)

**Table 3:** Percentage of patients who needed antiemetic therapy

Group	PACU (%)	2 hours (%)	24 hours (%)	Total (%)
1	15 (3/20)	30 (6/20)	0 (0/20)	35 (7/20)
2	10 (2/20)	40 (8/20)	30 (6/20)	60 (12/20)
3	5 (1/20)	55 (11/20)	20 (4/20)	70 (14/20)
4	5 (1/20)	5 (1/20)	15 (3/20)	25 (5/20)

**Table 4:** Percentage of patients who needed narcotic analgesics

Group	PACU (%)	2 hours (%)	24 hours (%)	Total number (%)
1	60 (12/20)	25 (5/20)	10 (2/20)	70 (14/20)
2	75 (5/20)	20 (4/20)	35 (7/20)	85 (17/20)
3	80 (16/20)	40 (8/20)	10 (2/20)	90 (18/20)
4	60 (12/20)	15 (3/20)	330 (6/20)	55 (11/20)

## DISCUSSION

This prospective, randomized, controlled trial has shown a reduced incidence of PONV using fluid supplementation (10 ml/kg) with LR, as compared to other fluid therapies (Hetastarch, Pentastarch, or Tetrastarch) in ASA grade I and II patients undergoing LC. The reduction in the incidence of PONV was more evident in the early (0-2 hours) postoperative period. However, 24 hours postoperatively, patients who had received fluid preloading with tetrastarch showed a significantly lower incidence of PONV compared to the other three groups. This could be explained by the longer effect of tetrastarch and its redistribution pattern compared to LR, and that in turn would provide tetrastarch as an adequate alternative to LR, for preventing PONV. The number of patients requiring rescue antiemetic was significantly less in the crystalloid group.

As a routine, during elective surgery, patients are advised to fast overnight. This combined with intraoperative anesthetic and surgical losses that are often inadequately replaced, results in hypovolemia with reduced blood flow to the gut. Gut ischemia if not corrected, is associated with excessive release of serotonin. Thus, fluid supplementation reduces the incidence of PONV, most probably, by improving the mesenteric perfusion and preventing gut ischemia and the resultant serotonin release.[9] Previously, numerous studies have considered different types of fluids such as colloids, crystalloids, or hypertonic solutions for perioperative fluid replacement. However, the amount of fluid administered has varied according to the type of volume replacement. Moreover, conclusions on the appropriate amount of fluid to administer obviously cannot be made from these studies.[10] In the postoperative period, avoidance of nausea in particular has been given high priority by this patient population.[11] The efficacy of the routine use of prophylactic antiemetics, remains controversial. Pharmacological prophylaxis has a limited effect, as measurable benefit is observed in only 20% of the patients receiving ondansetron to prevent PONV. Prophylactic antiemetic administration also increases the risk of adverse drug effects and side-effects, and increases the cost of care.[12] Crystalloid fluid administration may be a simple, inexpensive, non-pharmacological therapy that could reduce these symptoms, avoiding drug-related side-effects. The usefulness of multimodal therapy, particularly in high-risk cases, has been emphasized recently.[13] We have shown that the use of a fluid bolus as a preventive therapy is effective and may form an important part of multimodal prevention, while being cost-effective. Various studies have shown the incidence of ‘early PONV’ to be as high as 34% and the incidence of ‘late PONV’ to be 50%.[14] Earlier studies have also demonstrated the beneficial effect (reduction in PONV) of fluid supplementation.[15] It is worth mentioning here that we could demonstrate the beneficial effect of fluid therapy by using only 10 ml/kg of crystalloid, in comparison to 30 ml/kg of crystalloids used in another study.[16] Ali *et al*., also demonstrated a significant reduction in PONV, in patients who received 15 ml/kg of LR of fluid supplementation.[8] Although it is generally agreed that fluid therapy prevents PONV, not much work has been done on the type of the fluid. A literature search has revealed only one earlier study comparing the effect of crystalloids and colloids.[17] In that study colloid was used for intraoperative resuscitation in 90 patients undergoing elective non-cardiac surgery; they found that the incidence of nausea and vomiting, severe pain, periorbital edema, double vision, and the use of rescue antiemetics was significantly reduced in patients receiving colloids.[18] Maharaj *et al*., reported the use of large amounts of fluids during laparoscopic surgery, to decrease pain and PONV.[15] These data, despite being inconsistent, indicate that higher fluid amounts might reduce the risk of PONV and increase postoperative lung function after short operations. On the other hand, McCaul *et al*., showed that even a complete lack of any perioperative infusion did not increase the risk of PONV compared with infusing 1.1 L of LR.[7] In a recent published study, it was found that intravenous administration of LR solution at a dose of 30 ml/kg to patients undergoing thyroidectomy did not reduce the incidence of PONV, as also the antiemetic use when compared with LR at a dose of 10 ml/kg.[19] The impact of the type of fluid on PONV is still not well defined. In a recently published study the authors found that the type of fluid replacement administered (colloid vs. crystalloid) had minimal effect on the incidence of PONV.[20]

The current evidence suggests that liberal fluid is a good idea where major trauma and fluid shifting are unlikely, but more careful fluid management may be beneficial in more stressful operations.[21] In the present study, through the selection of patients and standardization of the anesthetic technique, we eliminated most of the risk factors which could lead to PONV and focused on the effect of only one variable, that is, type of fluid replacement.

## CONCLUSION

Preoperative fluid supplementation with LR, in a dose of 10 ml/kg, produced a lower incidence of PONV compared to colloid solutions. Tetrastarch could be a good alternative to LR, for prevention of PONV, due to its long lasting effect, up to 24 hours, postoperatively. Being non-pharmacological, we believe that preloading with LR solution can be carried out effectively in preventing early PONV following LC under general anesthesia.
